# Supplementary material for: Lipid nanoparticle-encapsulated Dnai1 mRNA rescues ciliary activity in primary ciliary dyskinesia mouse cell models
Source: J Cell Sci. 2025 Oct 27;138(20):jcs264068. doi: 10.1242/jcs.264068 (PMC12633742; doi:10.1242/jcs.264068)
Supplement: Supplementary information [file joces-138-264068-s1.pdf]

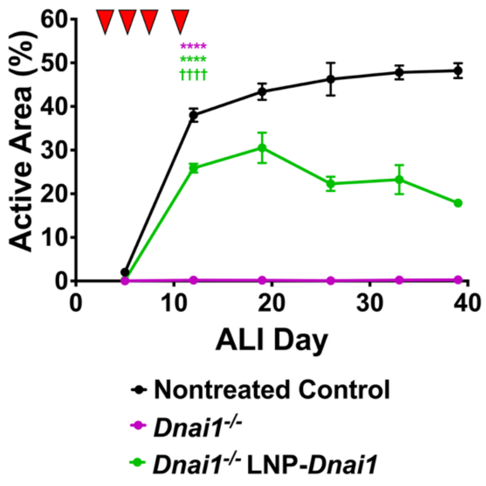

Fig. S1. Replicate study demonstrates the reproducibility for SORT-LNP-*Dnai1* treatment to rescue and maintain ciliary activity in *Dnai1*$^{-/-}$ mNPEC.

Active area measurements on differentiating *Dnai1*$^{-/-}$ mNPEC that were treated 3X/ week basolaterally with 10 µg/mL SORT-LNP-*Dnai1* on ALI days 4, 6, 8, and 11 (red arrows) for a total of four times. Data shown are mean ± s.d. This study was performed for two independent experiments in triplicate, with replicate data presented in Fig. 3. A one-way ANOVA followed by a Tukey's multiple comparison test suggests that the mean active area on ALI day 12 for the treated mNPEC was significantly different when compared to the nontreated control (****, $p < 0.0001$) and the *Dnai1*$^{-/-}$ mNPEC (††††, $p < 0.0001$).

Journal of Cell Science • Supplementary information

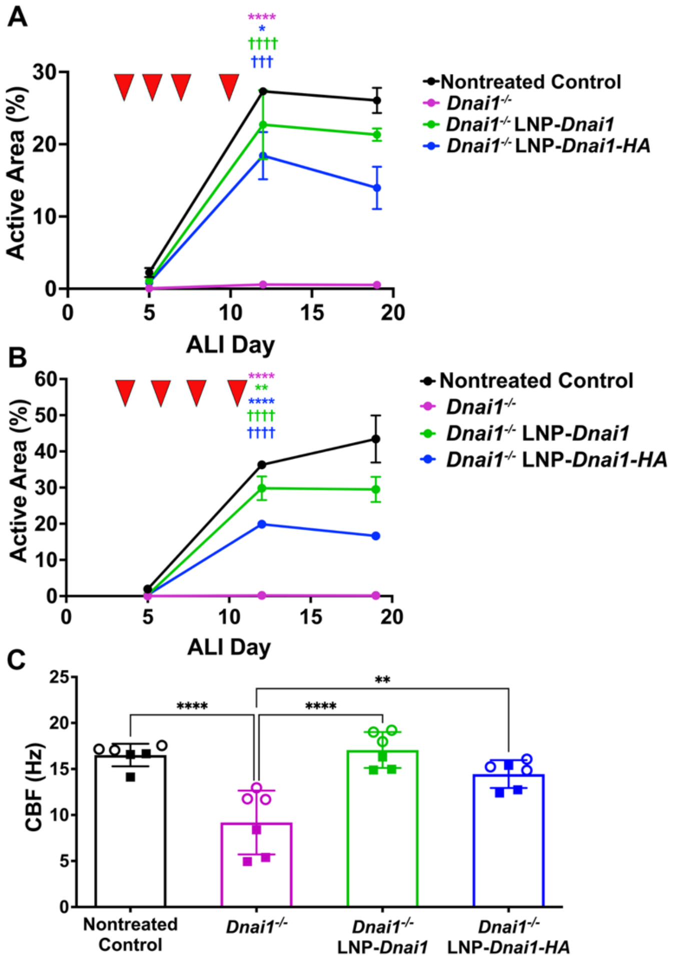

Fig. S2. Treatment with SORT-LNP containing carboxy-terminal HA-tagged *Dnai1* mRNA can rescue ciliary activity with a normal CBF.

Differentiating *Dnai1*$^{-/-}$ mNPEC were treated 3X/week basolaterally with 10 µg/mL SORT-LNP-mRNA for a total of four times starting at ALI Day 3 or 4. **A.)** Active area measurements on *Dnai1*$^{flox/flox}$ and *Dnai1*$^{-/-}$ mNPEC that were treated with 10 µg/mL SORT-LNP-mRNA on ALI days 3, 5, 7 and 10 (red arrows). **B.)** Active area measurements on differentiating *Dnai1*$^{\text{flox/flox}}$ and *Dnai1*$^{-/-}$ mNPEC that were treated with 10 µg/mL SORT-LNP-mRNA on ALI days 4, 6, 8, and 11 (red arrows). For panels **A** and **B**, data shown are mean ± s.d. for two independent experiments were performed in triplicate, where each replicate is represented in panel **A** and **B**, respectively. A one-way ANOVA followed by a Tukey's multiple comparison test suggests that the mean active area on ALI day 12 for the treated mNPEC was significantly different when compared to the nontreated control (*, $p < 0.05$; **, $p < 0.01$; ****, $p < 0.0001$) and the *Dnai1*$^{-/-}$ mNPEC (†††, $p < 0.001$; ††††, $p < 0.0001$). **C.)** Ciliary beat frequency (CBF) measurements on *Dnai1*$^{\text{flox/flox}}$ and *Dnai1*$^{-/-}$ mNPEC on ALI day 19. Data shown are mean ± s.d. from two independent experiments performed in triplicate. Open circle and square datapoints represent mean CBF from individual cultures from the first and second replicate experiment, respectively. An ordinary one-way ANOVA test with Tukey's multiple comparisons determined a significant difference between the mean CBFs (**, $p < 0.01$; ****, $p < 0.0001$).

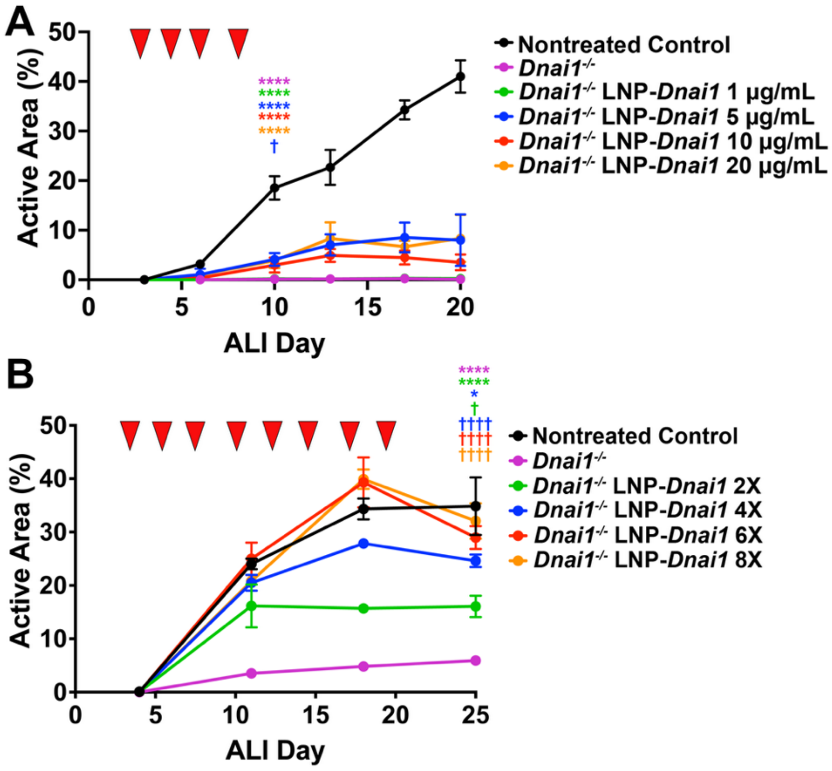

Fig. S3. Replicate study confirms six treatments of 5 µg/mL SORT-LNP-*Dnai1* at a frequency of 3x/week is the optimal dosage regimen. A.) Active area measurements on differentiating *Dnai1*$^{-/-}$ mNPEC cultures that were treated basolaterally with 1, 5, 10, or 20 µg/mL SORT-LNP-*Dnai1* 3X/week on ALI days 2, 4, 6, and 9 (red arrows) for a total of four times. Data shown are mean ± S.D from two independent experiments performed in triplicate, where the replicate is represented in Fig. 5C. **B.)** Active area measurements on differentiating Nontreated Control *Dnai1*$^{flox/flox}$ mNPEC, *Dnai1*$^{-/-}$ mNPEC (*Dnai1*$^{-/-}$), or *Dnai1*$^{-/-}$ mNPEC treated with 5 µg/mL SORT-LNP-*Dnai1* (*Dnai1*$^{-/-}$ LNP-*Dnai1*) 3X/week on ALI days 3, 5, 7, 10, 12, 14, 17, and 19 (red arrows) up to a total of eight times. Data shown are mean ± s.d. from two independent experiments performed in triplicate, with the replicate presented in Fig. 4F. For panels **A** and **B**, the mean active areas of treated mNPEC cultures on ALI day 10 or ALI day 25 were significantly different when compared to the nontreated control (*, $p < 0.05$; ****, $p < 0.0001$) and the *Dnai1*$^{-/-}$ mNPEC (†, $p < 0.05$; ††††, $p < 0.0001$) based on a one-way ANOVA followed by Tukey's multiple comparisons test.

Journal of Cell Science • Supplementary information

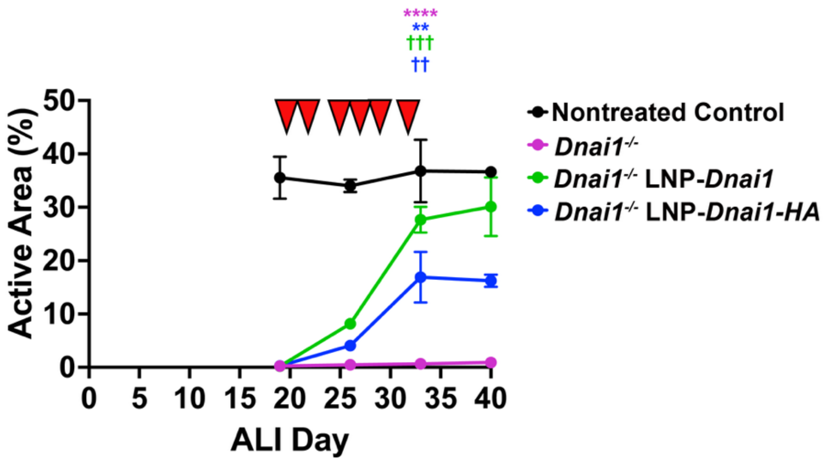

Fig. S4. Replicate study demonstrates the reproducibility of rescued ciliary activity in differentiated *Dnai1*$^{-/-}$ mNPEC with SORT-LNP-*Dnai1* treatment.

Active area measurements on fully differentiated *Dnai1*$^{-/-}$ mNPEC that were treated basolaterally with 5 µg/mL SORT-LNP 3X/week on ALI days 20, 22, 25, 27, 29, and 32 (red arrows) for a total of six times. Data shown are mean ± s.d. for two independent experiments performed in triplicate, with the replicate presented in Fig. 5C. Based on a one-way ANOVA with a Tukey's multiple comparison test, the mean active areas of treated mNPEC cultures on ALI day 34 were significantly different when compared to the nontreated control (**, $p < 0.01$; ****, $p < 0.0001$) and the *Dnai1*$^{-/-}$ mNPEC (††, $p < 0.01$; †††, $p < 0.001$).

Journal of Cell Science • Supplementary information

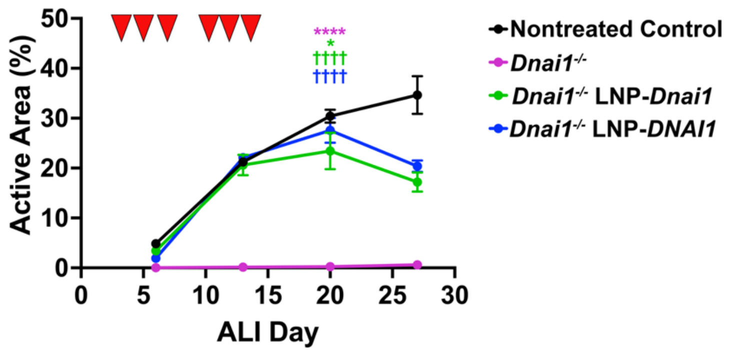

Fig. S5. Replicate study demonstrates ciliary activity can be restored in *Dnai1*$^{-/-}$ mNPEC through treatment with LNP-encapsulated human *DNAI1* mRNA.

Active area measurements on differentiating *Dnai1*$^{-/-}$ mNPEC that were treated basolaterally 3X/ week with 5 µg/mL SORT-LNP on ALI days 3, 5, 7, 10, 12, and 14 (red arrows) for a total of six times. Data shown are mean ± s.d. for two independent experiments performed in triplicate, where the replicate is presented in Fig. 6C. Based on a one-way ANOVA with a Tukey's multiple comparison test, the mean active areas of treated mNPEC cultures on ALI day 20 were significantly different when compared to the nontreated control (*, $p < 0.05$; ****, $p < 0.0001$) and the *Dnai1*$^{-/-}$ mNPEC (††††, $p < 0.0001$).

Journal of Cell Science • Supplementary information

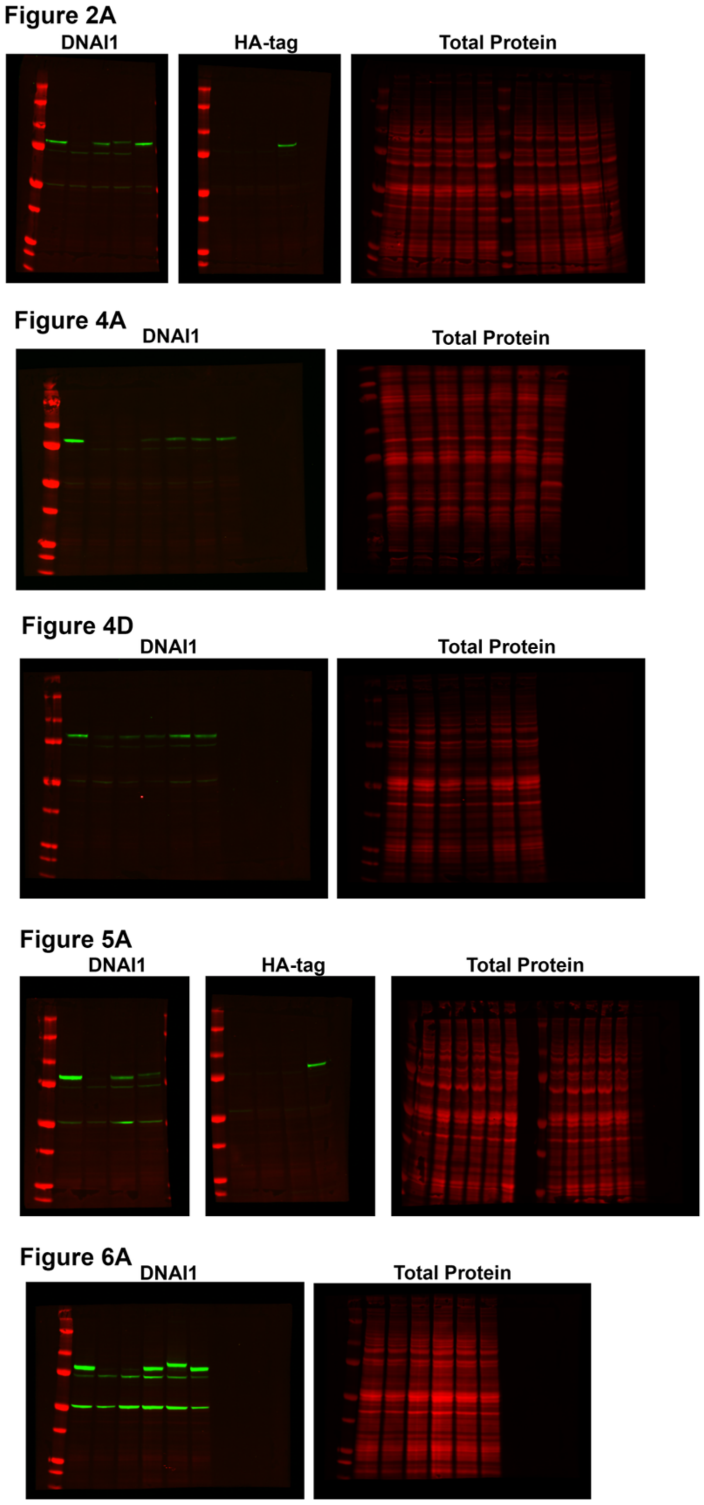

Fig. S6. Blot Transparency

Journal of Cell Science • Supplementary information

Table S1. LNP Formulation Characteristics

Formulation	Particle Size (nm)*	Polydispersity Index	Zeta Potential (mV)	Encapsulation Efficiency (%)	Nominal mRNA Concentration (mg/mL)
LNP-formulated Mouse *Dnai1* mRNA (ranges shown)	62 - 76	0.1	ND	94.0 – 98.2	0.77 – 1.08
LNP-formulated HA-tagged Mouse *Dnai1* mRNA (ranges shown)	67 - 76	0.1	ND	92.0 – 98.1	0.86 – 0.95
LNP-formulated Human *DNAI1* mRNA	70	0.1	ND	98.3	0.94
LNP-formulated HA-tagged Human *DNAI1* mRNA	69	0.1	1.81	93.8	1.07
LNP-formulated *tdTomato* mRNA	118	0.1	-0.230	48.1	0.50

*Particle size was measure by Malvern dynamic light scattering unit; ND; not determined.

Table S2. Primary and Secondary Antibodies Used

**Primary Antibodies**						
**Target**	**Host Species**	**Clonality**	**Source**	**Catalog Number**	**Immunoblot Dilution**	**Immunofluorescence Dilution**
DNAI1*	Mouse	Monoclonal	N/A	N/A	1:3,000	1:1,000
DNAI1	Rabbit	Polyclonal	Atlas Antibodies	HPA021649	1:1,000	1:500
HA-tag	Rabbit	Polyclonal	Sigma-Aldrich	H6908	1:1,000	1:500
Acetylated αTubulin	Mouse	Monoclonal	Sigma-Aldrich	T7451	N/A	1:500
**Secondary Antibodies**						
**Target**	**Host Species**	**Clonality**	**Source**	**Catalog Number**	**Immunoblot Dilution**	**Immunofluorescence Dilution**
αMouse IRDye 680RD	Goat	Polyclonal	LI-COR	925-68070	1:7,500	N/A
αRabbit IRDye 800CW	Donkey	Polyclonal	LI-COR	925-32213	1:7,500	N/A
αMouse Alexa Fluor 488	Donkey	Polyclonal	Thermo Fisher	A-21202	N/A	1:1,000
αRabbit Rhodamine Red X	Donkey	Polyclonal	Jackson Immuno	711-296-152	N/A	1:1,000
αRabbit Alexa Fluor 647	Donkey	Polyclonal	Thermo Fisher	A-31573	N/A	1:1,000
Hoechst33342 (DNA Dye)	N/A	N/A	Thermo Fisher	H1399	N/A	1:500

*DNAI1 monoclonal antibody raised in mouse was previously generated by Ostrowski *et al*.

Journal of Cell Science • Supplementary information

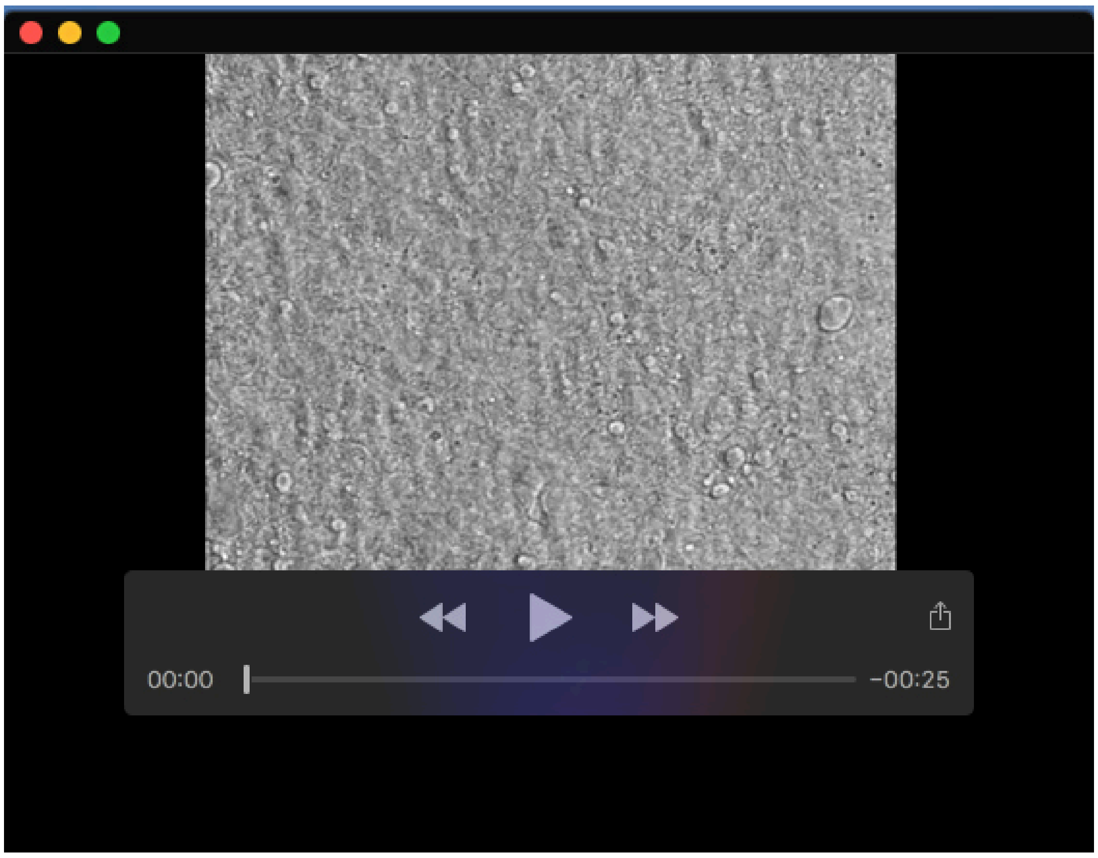

Movie 1. Top-down view of whole field of Nontreated Control *Dnai1*$^{flox/flox}$ mNPEC Representative video of whole field of view of Nontreated Control *Dnai1*$^{flox/flox}$ mNPEC that demonstrates a high level of ciliary activity with a range of ciliary beat frequencies. A heat map showing the range of ciliary beat frequencies corresponding with this video is presented in Fig. 3C. Videos were taken by SAVA software on a Nikon Eclipse TE2000 inverted microscope with a 20X objective at 37°C and 5% CO_2 with humidified air.

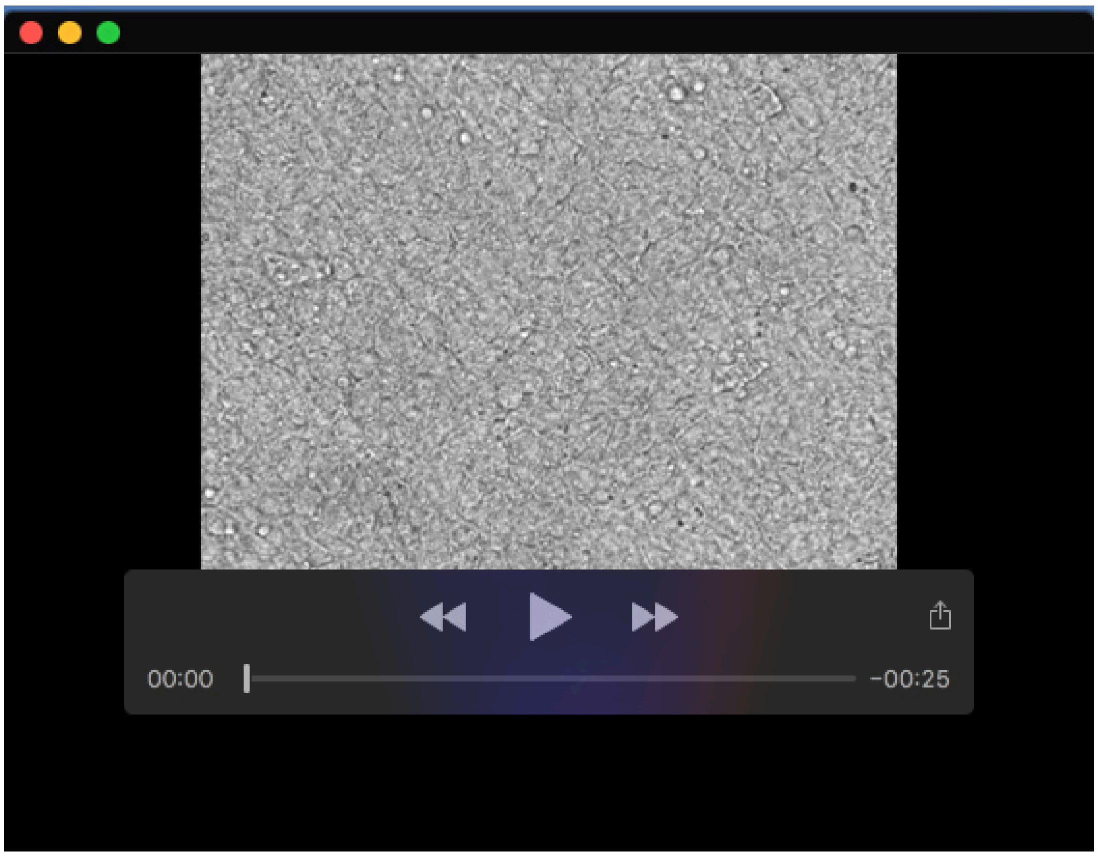

Movie 2. Top-down view of whole field of *Dnai1*$^{-/-}$ mNPEC Representative video of whole field of view of *Dnai1*$^{-/-}$ mNPEC showed miniscule levels of ciliary activity with few active ciliated cells that exhibited a low ciliary beat frequency. A heat map showing the limited range of ciliary beat frequencies corresponding with this video is presented in Fig. 3C. Videos were taken by SAVA software on a Nikon Eclipse TE2000 inverted microscope with a 20X objective at 37°C and 5% CO_2 with humidified air.

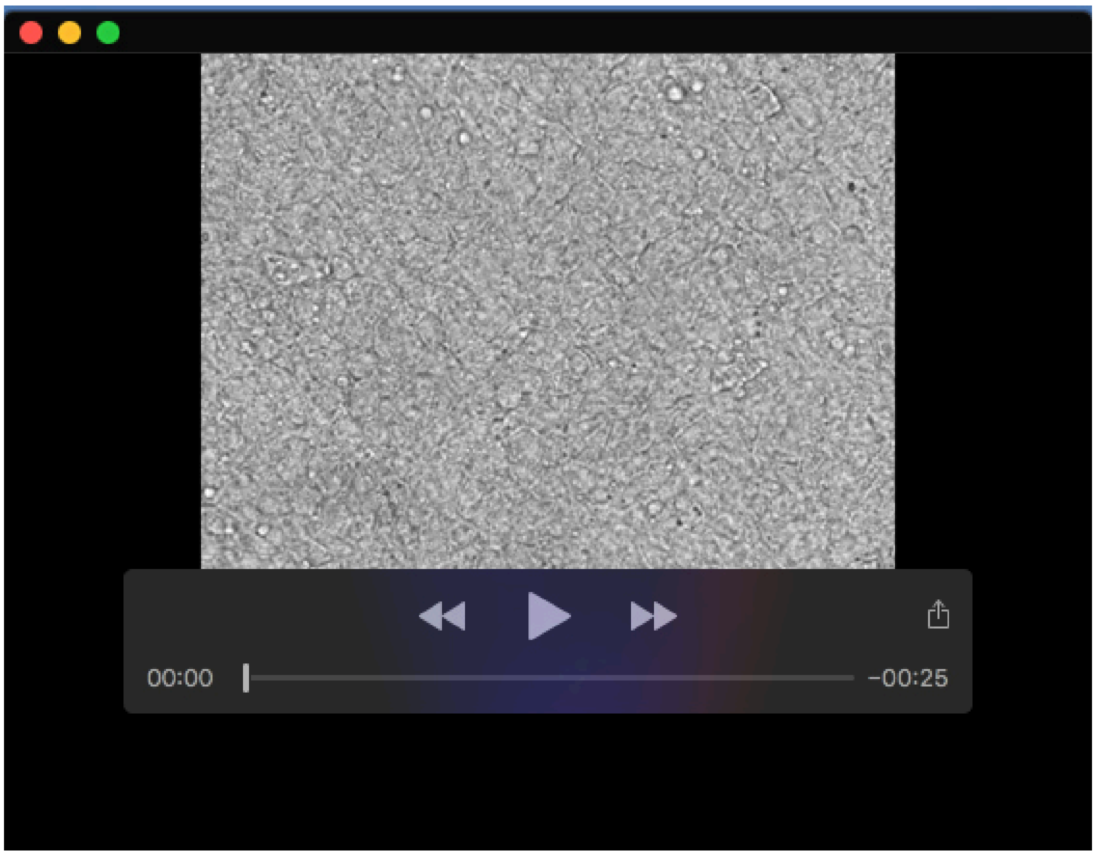

Movie 3. Top-down view of whole field of *Dnai1*$^{-/-}$ mNPEC treated with SORT-LNP-*Dnai1* Representative video of whole field of view of SORT-LNP-*Dnai1*-treated *Dnai1*$^{-/-}$ mNPEC showed high levels of rescued ciliary activity with a range of ciliary beat frequencies that reached normal levels. A heat map showing the range of ciliary beat frequencies corresponding with this video is presented in Fig. 3C. Videos were taken by SAVA software on a Nikon Eclipse TE2000 inverted microscope with a 20X objective at 37°C and 5% CO_2 with humidified air.

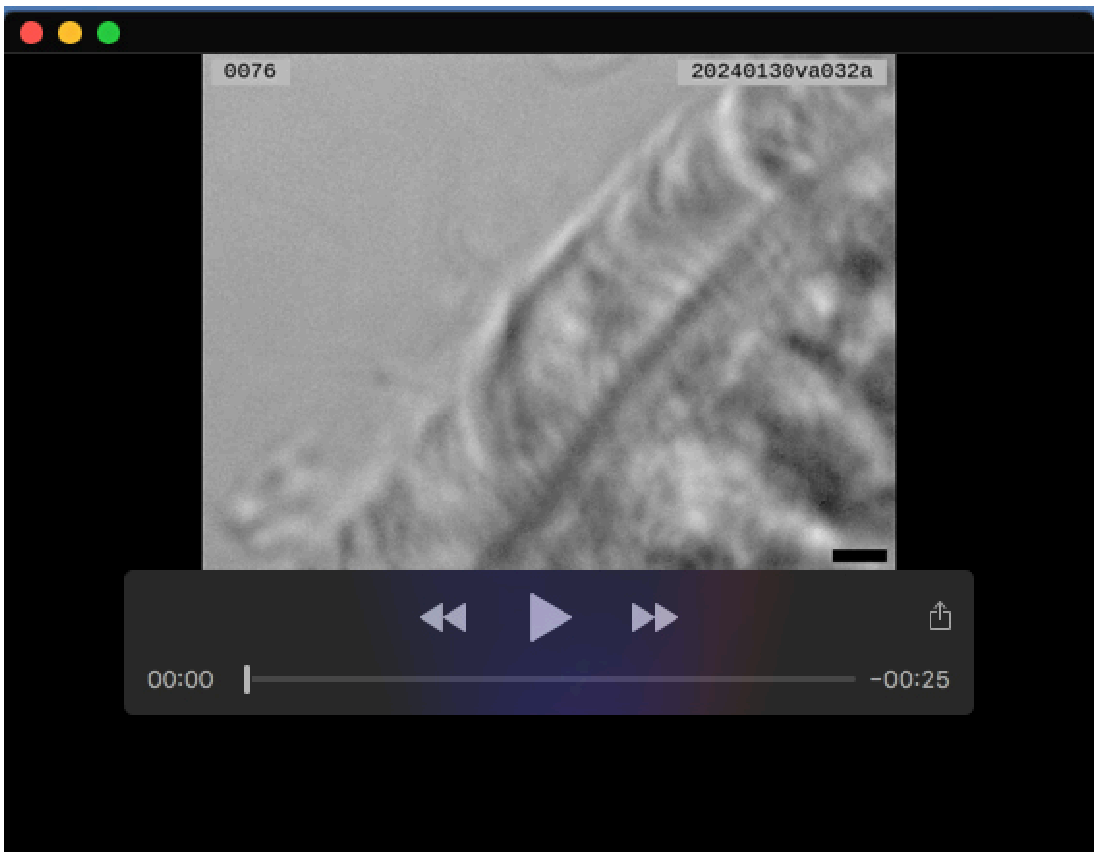

Movie 4. High-resolution profile view of motile cilia from Nontreated Control *Dnai1*$^{flox/flox}$ mNPEC Representative high-resolution video of a profile view of active motile cilia from Nontreated Control *Dnai1*$^{flox/flox}$ murine nasopharyngeal epithelial cells scraped from cultures showing normal waveform at a replay speed of 30 fr/s (0.1 x live). Videos of scraped cells were taken by SAVA software on a Nikon Eclipse TE2000 inverted microscope with a 60X oil objective (NA=1.4) with DIC-H optics and 2X post-objective magnification at 37°C and 5% CO_2 with humidified air. Scale bar, 2 µm.

Journal of Cell Science • Supplementary information

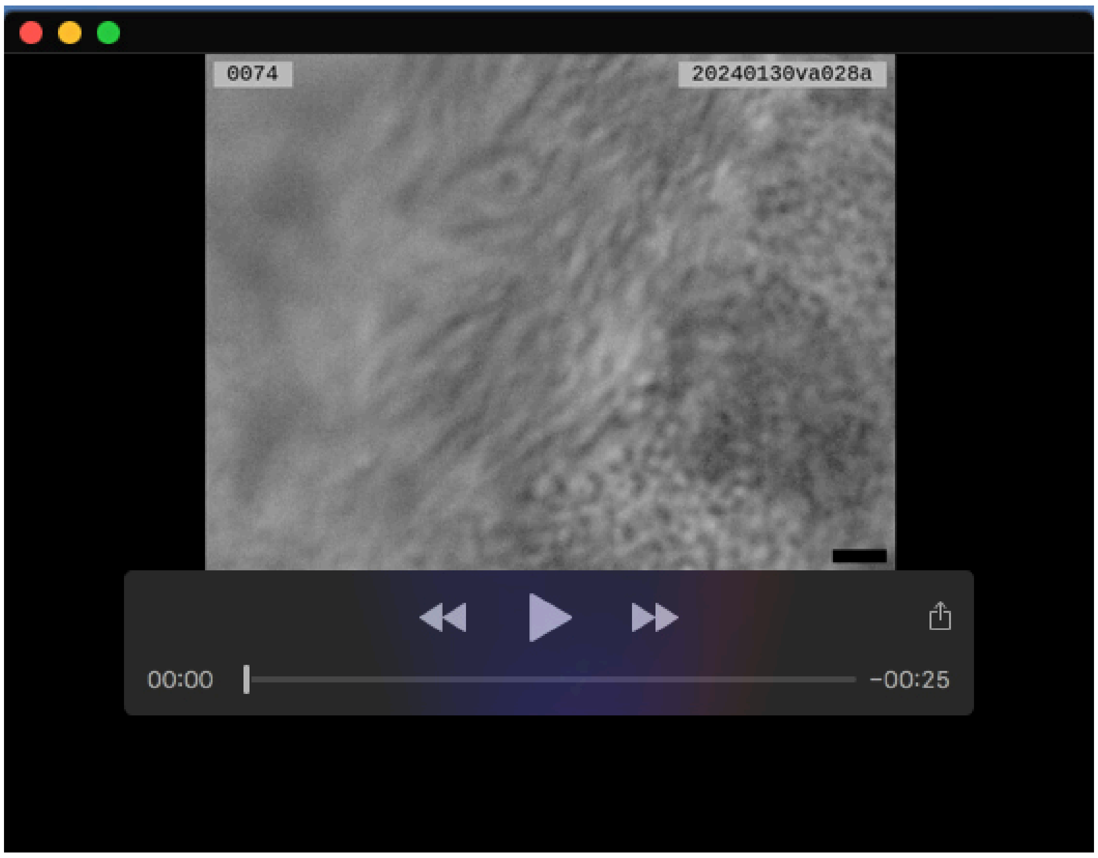

Movie 5. High-resolution top-down view of motile cilia from Nontreated Control *Dnai1*$^{flox/}$$_{flox}$ mNPEC

Representative high-resolution video of a top-down view of active motile cilia from Nontreated Control *Dnai1*$^{flox/flox}$ murine nasopharyngeal epithelial cells scraped from cultures showing normal ciliary coordination at a replay speed of 30 fr/s (0.1 x live). Videos of scraped cells were taken by SAVA software on a Nikon Eclipse TE2000 inverted microscope with a 60X oil objective (NA=1.4) with DIC-H optics and 2X post-objective magnification at 37°C and 5% CO_2 with humidified air. Scale bar, 2 µm.

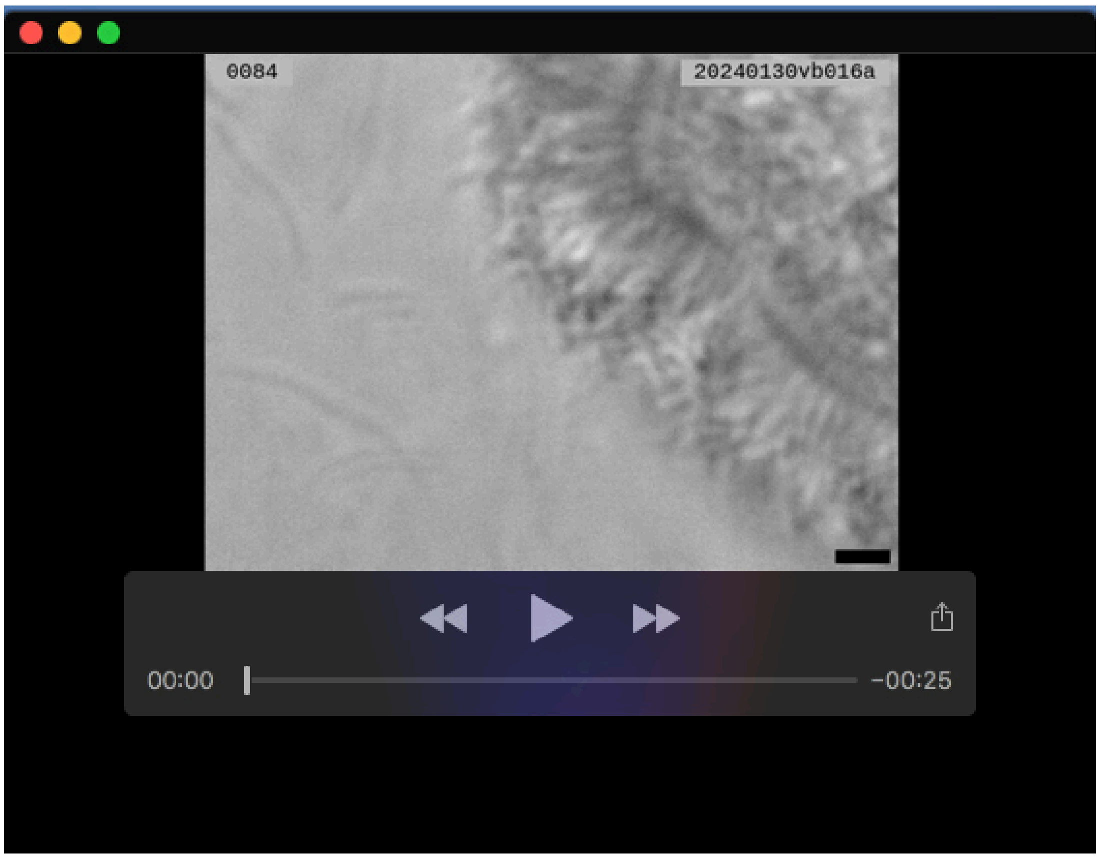

Movie 6. High-resolution profile view of motile cilia from *Dnai1*$^{-/-}$ mNPEC Representative high-resolution video of a profile view of motile cilia from *Dnai1*$^{-/-}$ murine nasopharyngeal epithelial cells scraped from cultures at a replay speed of 30 fr/s (0.1 x live) showing few active cilia with a lower CBF. Videos of scraped cells were taken by SAVA software on a Nikon Eclipse TE2000 inverted microscope with a 60X oil objective (NA = 1.4) with DIC-H optics and 2X post-objective magnification at 37°C and 5% CO_2 with humidified air. Scale bar, 2 µm.

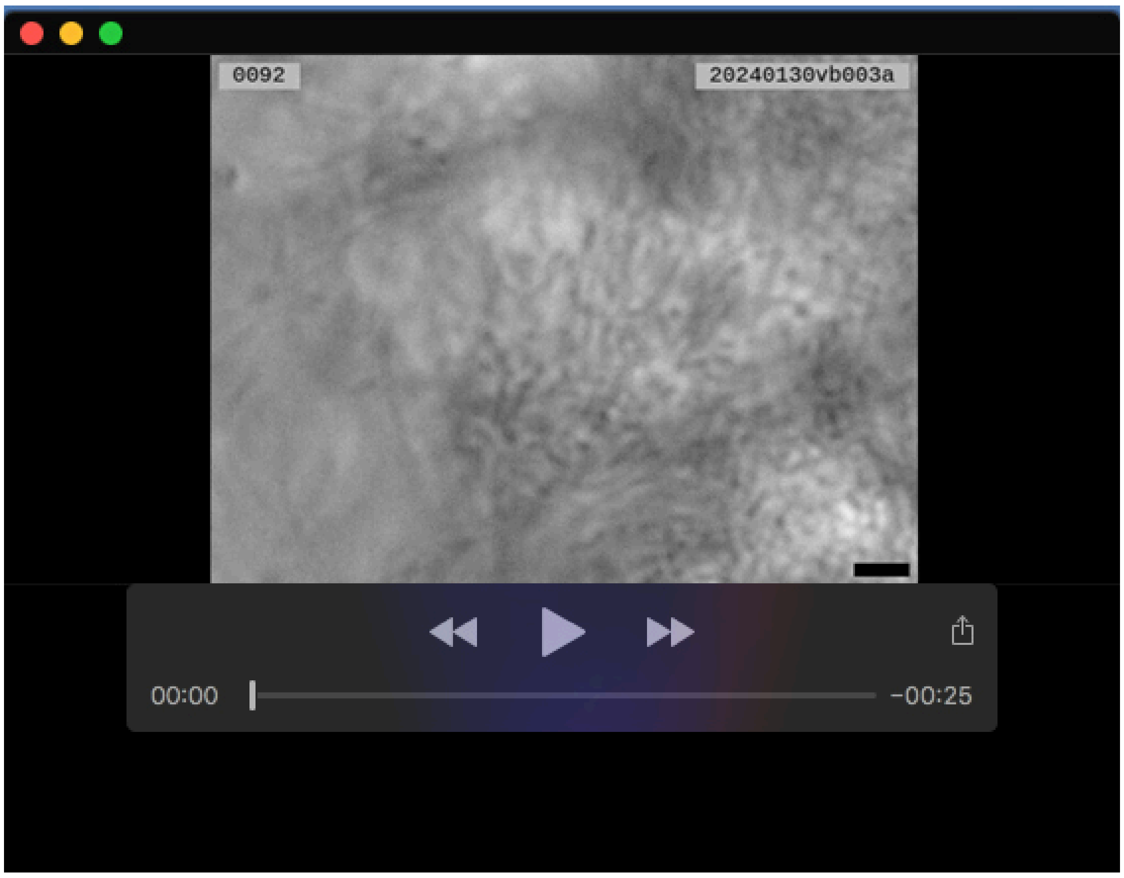

Movie 7. High-resolution top-down view of motile cilia from *Dnai1*$^{-/-}$ mNPEC Representative high-resolution video of a top-down view of motile cilia from *Dnai1*$^{-/-}$ murine nasopharyngeal epithelial cells scraped from cultures at a replay speed of 30 fr/s (0.1 x live) showing few active cilia that are uncoordinated. Videos of scraped cells were taken by SAVA software on a Nikon Eclipse TE2000 inverted microscope with a 60X oil objective (NA = 1.4) with DIC-H optics and 2X post-objective magnification at 37°C and 5% CO_2 with humidified air. Scale bar, 2 μm.

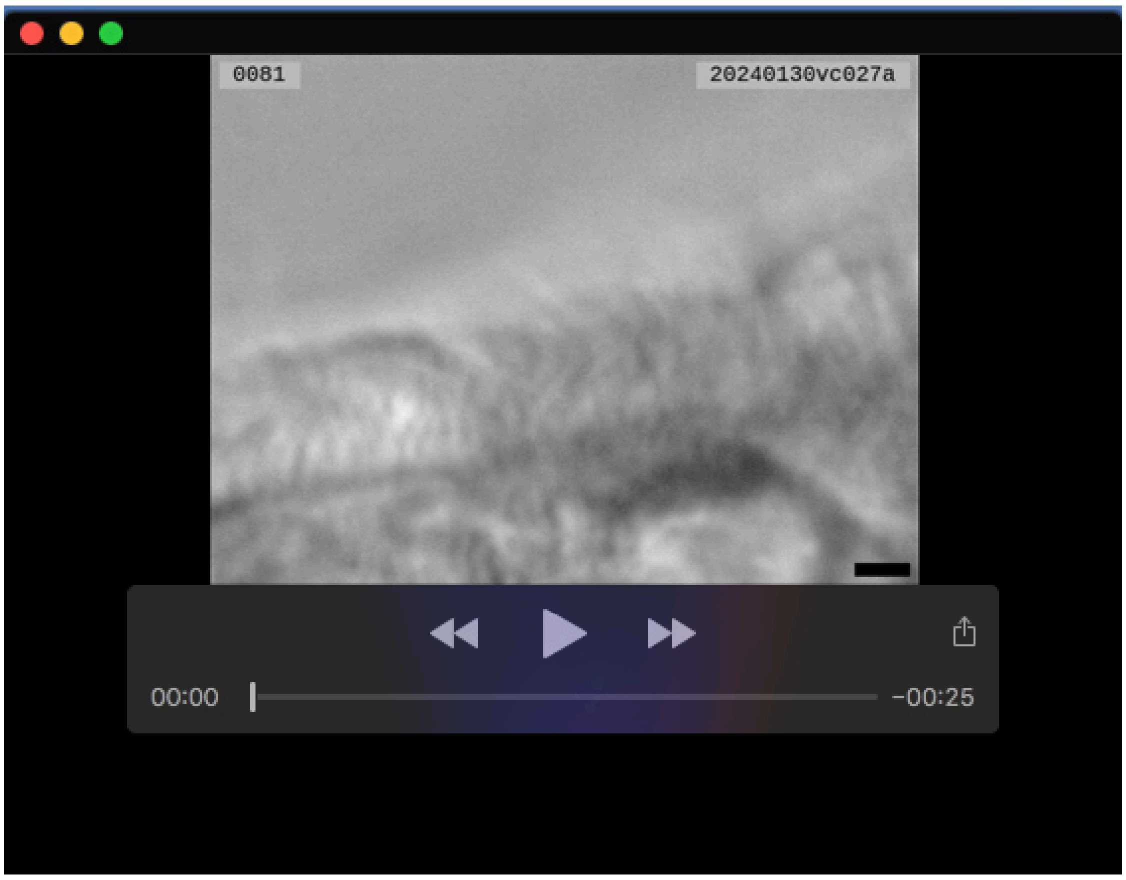

Movie 8. High-resolution profile view of motile cilia from *Dnai1*$^{-/-}$ mNPEC treated with SORT-LNP-*Dnai1* Representative high-resolution video of a profile view of motile cilia from *Dnai1*$^{-/-}$ murine nasopharyngeal epithelial cells treated with SORT-LNP-*Dnai1* scraped from cultures at a replay speed of 30 fr/s (0.1 x live) showing active cilia with a normal waveform. Videos of scraped cells were taken by SAVA software on a Nikon Eclipse TE2000 inverted microscope with a 60X oil objective (NA = 1.4) with DIC-H optics and 2X post-objective magnification at 37°C and 5% CO_2 with humidified air. Scale bar, 2 μm.

Journal of Cell Science • Supplementary information

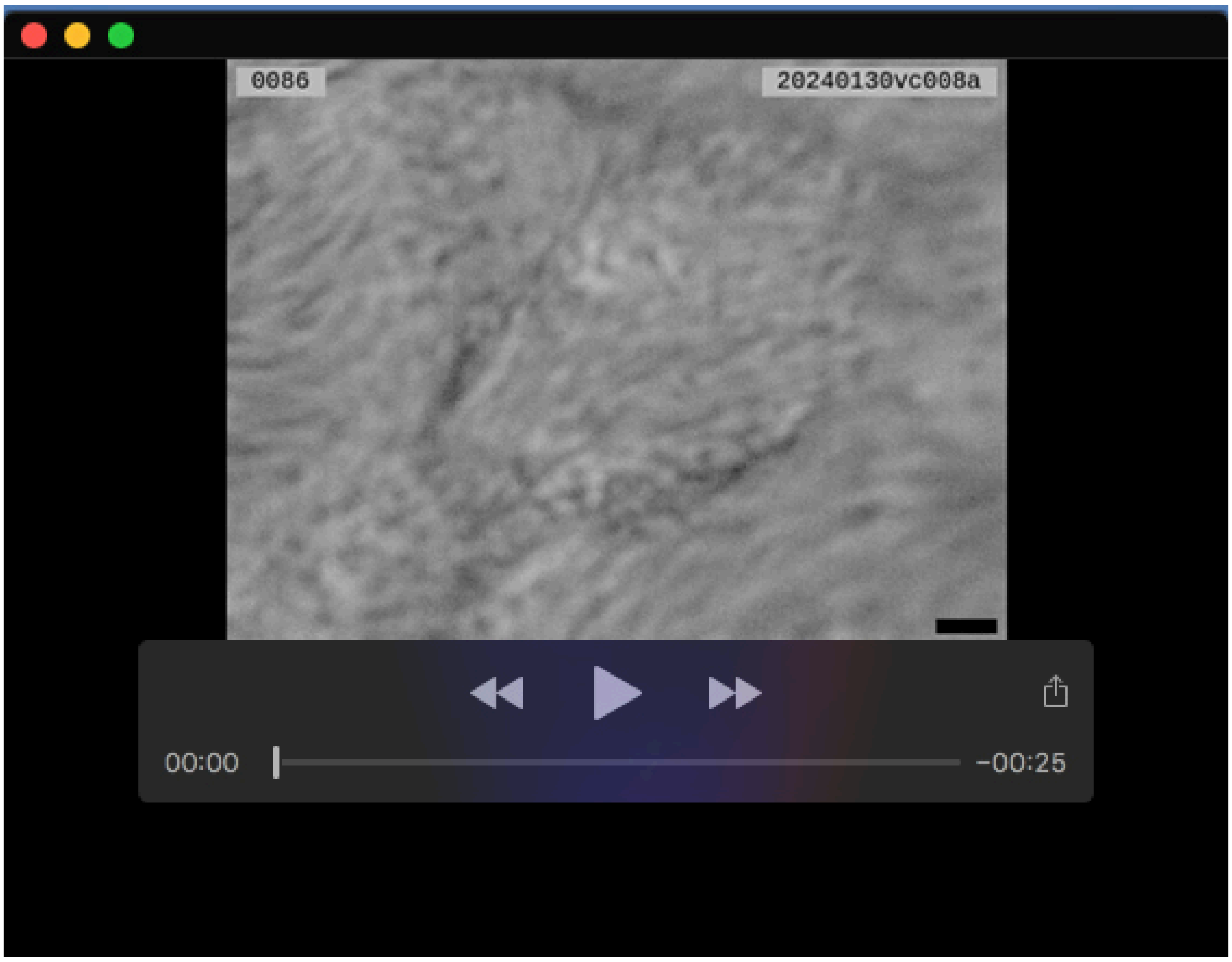

Movie 9. High-resolution top-down view of motile cilia from *Dnai1*$^{-/-}$ mNPEC treated with SORT-LNP-*Dnai1*
Representative high-resolution video of a top-down view of motile cilia from *Dnai1*$^{-/-}$ murine nasopharyngeal epithelial cells treated with SORT-LNP-*Dnai1* scraped from cultures at a replay speed of 30 fr/s (0.1 x live) showing active motile cilia that are, to a degree, coordinated. Videos of scraped cells were taken by SAVA software on a Nikon Eclipse TE2000 inverted microscope with a 60X oil objective (NA = 1.4) with DIC-H optics and 2X post-objective magnification at 37°C and 5% CO_2 with humidified air. Scale bar, 2 µm.

Journal of Cell Science • Supplementary information
